# Knowledge, attitude and practices of school teachers towards epileptic school children in Karachi, Pakistan

**DOI:** 10.12669/pjms.301.4307

**Published:** 2014

**Authors:** Nasha Homi Bhesania, Anaya Rehman, Ilma Saleh Savul, Nosheen Zehra

**Affiliations:** 1 Nasha Homi Bhesania, Fourth Year MBBS Students, Ziauddin University, Karachi, Pakistan.; 2 Anaya Rehman, Fourth Year MBBS Students, Ziauddin University, Karachi, Pakistan.; 3 Ilma Saleh Savul, Fourth Year MBBS Students, Ziauddin University, Karachi, Pakistan.; 4 Nosheen Zehra, Assistant Professor, Department of Community Health Sciences, Ziauddin University, Karachi, Pakistan.

**Keywords:** Epilepsy, Knowledge, Attitude, School teachers

## Abstract

***Objective:*** To assess the knowledge and attitude of school teachers towards an epileptic child.

***Methods:*** This cross-sectional study was conducted among teachers of primary, middle and high schools from different communities in Karachi, Pakistan. A total of 120 self-administered KAP questionnaires were distributed in schools selected through convenience sampling. Data was analyzed using SPSS version 19.

***Results:*** Out of the total sample size, 90.9% (n=100) had heard about epilepsy. Sixteen (14.5%) teachers considered epilepsy to be contagious while 72.7% (n=80) teachers were of the view that epileptics can succeed as other normal children and sixty nine (62.7%) teachers were willing to help a child experiencing a fit, though only 15.5% (n=17) received knowledge about epilepsy during their training years.

***Conclusion:*** The research was conclusive for assessing the knowledge and attitude of teachers towards epileptic children. It highlighted the lack of awareness and negative attitude still existing among teachers. However, many were in the favor of mass education through awareness programs.

## INTRODUCTION

Among non communicable diseases epilepsy is a chronic disorder of the brain that occurs worldwide affecting people of all age groups. It refers to a clinical phenomenon rather than a single disease entity, characterized by recurrent seizures. Currently, epilepsy affects 50 million people worldwide, and 80% of them live in the developing world.^1^ Prevalence of epilepsy in Pakistan has been under study. In 1994 it was prevalent in 1% of Pakistani population,^2^ whereas it was estimated to be 9.99 per 1000 population in 2003.^3^

It is unfortunate that a common disease like epilepsy carries strong social stigma. The social attitude leading to stigma and discrimination against epileptics is often more distressing than the disease itself.^4^ Teachers’ attitude based on their knowledge and beliefs plays an immense role in the upbringing of epileptic children. Misbeliefs cause social discrimination against epileptics^5^ since childhood. Hence the teacher’s knowledge regarding epilepsy is an important element of the educational experiences of epileptics.

As in other parts of the world, teachers in Pakistan also have a key role in society as educators. In 2007 a study conducted in slum area of Karachi revealed significant gaps in the community’s knowledge on epilepsy.^6^ Hence, the aim of this study was to assess the knowledge about epilepsy among teachers in Karachi, their perceptions on how it affects the educational abilities of school children, their tendency to accept or reject the epileptic child and their ability to help a convulsing child.

## METHODS

This cross-sectional study was conducted among 120 teachers for a period of 4 months, from 1^st^ Nov, 2011 to 1^st^ March, 2012. Three schools were randomly mapped in Karachi by convenience sampling and self-administered questionnaires were distributed amongst teachers. A verbal informed consent was obtained from the teachers and each one was asked to fill the questionnaire in isolation. No attempt was made to prompt the responder. 

The questionnaire was constructed after a careful review of relevant literature^1^^,^^3^,^7^^-^^9^ and pre-tested in pilot study. It was divided into three sections; Section A based on teachers’ demographic data and knowledge, Section B on their attitude toward epileptics and questions regarding practices were part of Section C. All questions were close ended with ‘Yes’ ‘No’ or ‘I don’t know’ responses.

Data was analyzed using the Statistical Program for Social Sciences (SPSS, version 19). All quantitative variables were presented as mean and standard deviation and all qualitative variables were presented as percentages and frequencies. 

## RESULTS

A total of 120 questionnaires were distributed in three schools of Karachi of which 110 were returned and included in the data, giving a response rate of 91.7%. Most of the questions were answered, few were left unanswered. The mean age of the total sample was 37.70 ± 11.77 years and that of males and females was calculated 36.56 ± 10.92 and 38.26 ± 12.19 years respectively. The percentages and frequencies of respondents were 32.7% (n=36) males and 67.3% (n=74) females. Of all, 48.2% (n=53) of respondents comprised of high school teachers, 23.6% (n=26) were middle school teachers, 20% (n=22) were primary school teachers and 8.2% (n=9) were kindergarten teachers. Overall 50% (n=55) teachers had teaching experience of more than 10 years.

Amongst all, 90.9% (n=100) heard about epilepsy, with print media 60.9% (n=40) being the commonest source of information, followed by friends and family 50.9% (n=56), electronic media 25.5% (n=28) and doctor 13.6% (n=15). Only 9 (8.2%) respondents had no knowledge of the disorder at the time of filling the questionnaire. Knowledge of teachers regarding different aspects of epilepsy is presented in Table-I.

**Table-I:** Knowledge of teachers regarding epilepsy (N= 120).

*Signs & symptoms of epilepsy*	*n*	*%*
Fainting	90	81.8
Staring blankly into space	56	50.9
Blinking of eyes and jerks	70	63.6
Tongue rolling back	67	60.9
*Causes of epilepsy*		
Head injury	43	39.1
Brain stroke	56	50.9
Brain infection	21	19.1
Without specific cause	30	27.3
*Myths/beliefs about epilepsy*		
A contagious disease	16	14.5
Causes mental retardation	38	34.5
A punishment for sins	11	10.0
Bewitchment / supernatural possession	12	10.9
*Barriers/problems faced by epileptic child*		
Labeled as a disabled child	42	38.2
Unable to get good education	45	40.9
Socially unaccepted	41	37.3
Suffer from low self esteem	67	60.9

According to 28.2% (n=31) teachers, epileptics have delayed growth and development with 27.3% (n=30) of the opinion that they cannot succeed as well as normal children. Regarding academic achievements of epileptics, 58.2% (n=64) teachers were of the opinion that it is hampered by the social stigmata and 59.1% (n=65) said it is due to disease itself. Suitable career options for epileptic children as suggested by teachers are presented in Table-II.

**Table-II:** Teachers’ choices of suitable careers for epileptics (N = 120).

*Career*	*n *	*%*
Teacher	61	50.9
Doctor	58	48.3
Accountant	53	44.5
Lawyer	46	38.2
Engineer	34	28.2
Sports	29	24.5
Pilot	4	3.6

Sixty seven (60.9%) teachers were of the positive opinion that epileptics should be allowed to engage in outdoor activities and 31.8% (n=35) prefer special schools for epileptic children. Fifty eight (52.7%) of the respondents said epilepsy is preventable but only 48.2% (n=53) said it is curable. The treatment options recommended were medications, traditional healers and God’s help/prayers by 84.5% (n=93), 20% (n=22) and 58.2% (n=64) teachers respectively.

Twenty nine (26.4%) of the teachers had witnessed a student in class having a seizure and 31.8% (n=35) knew someone with epilepsy. Five teachers (4.5%) refused to teach in a class with an epileptic child. Sixty nine (62.7%) teachers were willing to help if they witnessed a child having seizure and the frequently selected options to help a seizing child are highlighted in Table-III.

**Table-III:** Helping a seizing child (N = 120).

*Option to help a seizing child by*	*n *	*%*
Roll the child to side	64	58.2
Loosen clothing	50	45.5
Prevent tongue rolling back	50	45.5
Remove sharps from vicinity	67	60.9
Ensure breathing	72	65.5
Record duration of fit	56	50.9

Seventeen (15.5%) teachers had received knowledge about epilepsy in their training. Ninety (81.8%) teachers thought public awareness programs should be held at hospitals in Karachi and eighty (72.7%) teachers were of the opinion that such programs should also be incorporated in teachers training.

## DISCUSSION

Teachers have the key role as educators and advisors in any society; hence the study was aimed to assess the knowledge, attitude and practices of teachers towards epilepsy. Knowledge about epilepsy is shown to be high in several studies from developed as well as developing countries.^9^ Despite that, misconceptions have been associated with epilepsy since ancient times causing a great amount of stigma and discrimination against its sufferers. Previous studies have shown the prevalence of incorrect perceptions on epilepsy and negative attitude towards epileptics among teachers even in resource-rich countries.^10^ The magnitude of epilepsy stigma in limited-resource countries has not been fully estimated previously^11^^,^^12^ however, in our study majority of teachers identified the signs/symptoms and causes of epilepsy correctly, which shows that the teacher witnessing a child having seizures will consider it to be epilepsy and not any supernatural possession which is often a misconception.

This study, although small, has several important findings regarding teachers’ concepts. Although 90.9% had knowledge, it was surprising to see that 28.2% thought epileptics have delayed growth and development. This misconception was present even in those teachers with greater years of teaching experience. Furthermore, 27.3% said that epileptics cannot succeed as properly as children without epilepsy. These findings are important because the negative attitude of teachers will have a negative impact on students’ development. A recently published Iranian study on the attitude of biology teachers towards epileptics showed that fear, avoidance and other negative attitudes were observed among teachers.^13^ Another study, on the rural population of northeastern Thailand showed that 90% participants thought that epileptics cannot live in the society like healthy people do.^14^ Our study showed similar results, where 14.5% teachers thought epilepsy is contagious and 4.5% refused to teach epileptic children. 

Misconceptions arise due to myths and beliefs passing down generations. A number of them were identified, the commonest being that epilepsy causes mental retardation. The relation of epilepsy and insanity seems to vary amongst the developing countries. 23.6% of Vietnamese^15^ felt a direct relation between the two, only 16% of Chinese^16^ and 7% of Taiwanese^17^ felt the same. However, countries of the developed world namely USA^18^, Denmark^19^ and Italy^20^, have reported negative association between insanity and epilepsy. Misbelief, misguidance, and improper treatment in developing countries seem to be possible reasons for the difference in observations. The percentage of our teachers blaming epilepsy to be a form of supernatural possession (10.9%) is comparable to figures obtained from a similar Indian study (5.5%).^5^ This is high amongst teachers of the subcontinent as compared to those in Thailand (0.9%).^21^

Epileptic children are an educationally vulnerable group and the education staff needs to be mindful of the additional support that they may require. The barriers faced by epileptic children, as identified by this research are highlighted here for comparison to a previously conducted local study on the stigma of epilepsy.^22^ It stated that the students’ education and grades are affected by the disorder. Our study also showed that majority thought that an epileptic child’s academic achievement is hampered by the disease stigma and that special schools are preferred for such children. Perhaps, this was due to the ignorance of the additional support such children require, that they considered special schools as more appropriate. On the other hand it was encouraging to know that a large percentage of teachers said that epileptics should be allowed to engage in outdoor activities like other children. The choice of a suitable profession for epileptics, as chosen by majority was teaching and medicine. This result raises a point that if teachers were of the opinion that epileptics cannot succeed as well as other children, their choice of challenging and competent professions for epileptics remains to be questioned further.

A lot of variation is seen in the percentage of teachers believing epilepsy to be curable or not. In a study conducted among Italian school teachers, it was observed that 46.8% of the teachers believed that epilepsy was incurable,^23^ and a study in Brazil showed a much greater percentage of teachers believing epilepsy to be treatable (90%).^24^ A study conducted among Zambian teachers concluded that individuals with poorer knowledge were more likely to recommend traditional healers rather than physicians for treatment of epilepsy.^25^ Fortunately, 84.5% of our teachers recommended medication as a treatment modality.

In a survey conducted in Senegal, it was observed that a significant proportion of teachers mentioned harmful measures to manage a child having seizures in the classroom.^26^ In this study, the most frequently selected option among ways to help such childtren was by ensuring breathing. At the same time, preventing the tongue rolling back that indirectly fulfills the former purpose received the least positive responses, hence putting the ignorance of teachers under the spotlight.

The negative attitudes towards epileptics can be improved by awareness of the disease. Previous studies have pointed out the teachers’ eagerness to know about epilepsy.^26^^,^^27^ Here, most were of the opinion that knowledge should be incorporated into the teachers training by organizing awareness programs.

***Limitation and Recommendation: ***Public awareness on epilepsy is of great importance in improving the quality of life of epileptic children. The target population of this study is the teachers of Karachi, which is the largest city of Sindh. Due to limitation of resources a large number of sample was not captured in this study. Hence these results cannot be generalized to assess the knowledge of teachers throughout Pakistan. However, we recommend that this and other similar researches conducted in different cities and rural areas of Pakistan can be used to organize awareness programs in those regions where the lack of knowledge is most prevalent.

## CONCLUSION

Despite its limitations, the research was conclusive in bringing to light the lack of awareness and the negative attitude towards epilepsy still existing among teachers. The positive aspect was that many were in the favor of mass education programs. Better informed teachers are likely to have a more positive attitude, hence improving the management of epilepsy.

## Notes

***Source of Funding:*** None.
